# Clinical Effectiveness of Penicillin-Free Therapies in First-Line and Rescue Treatments for *Helicobacter pylori*: A Systematic Review

**DOI:** 10.3390/antibiotics14050476

**Published:** 2025-05-08

**Authors:** Kenza El Boury, Hind Boudarf, Imane Adoud, Soukaina Ouannass, Oussama Abi, Hanane Delsa, Fatima Azzahra Lahlou, Samy Iskandar, Meryem El Jemli, Idrissa Diawara, Mohamed Amine Senhaji, Lhousaine Balouch, Zakaria Belrhiti, Mohamed Kettani Halabi

**Affiliations:** 1 Research Laboratory in Drug Sciences, Mohammed VI Faculty of Pharmacy, Mohammed VI University of Sciences and Health, Casablanca 82403, Morocco; kelboury2@um6ss.ma (K.E.B.); hboudarf@um6ss.ma (H.B.); imaneadoud96@gmail.com (I.A.); souannass@um6ss.ma (S.O.); oabi@um6ss.ma (O.A.); siskandar@um6ss.ma (S.I.); meljemli@um6ss.ma (M.E.J.); lbalouch@um6ss.ma (L.B.); 2 Research Unit, Mohammed VI Center for Research and Innovation, Rabat 10112, Morocco; idiawara@um6ss.ma (I.D.); zbelrhiti@um6ss.ma (Z.B.); 3 Mohammed VI Faculty of Medicine, Mohammed VI University of Sciences and Health, Casablanca 82403, Morocco; hdelsa@um6ss.ma (H.D.); fzlahlou@um6ss.ma (F.A.L.); 4 Laboratory of Microbial Biotechnology and Infectiology Research, Mohammed VI University of Sciences and Health, Casablanca 82403, Morocco; 5 Department of Pharmacology and Physiology, Faculty of Medicine, University of Montreal, Montreal, QC H3T 1J4, Canada; mohamed.amine.senhaji@umontreal.ca; 6 Mohammed VI International School of Public Health, Mohammed VI University of Sciences and Health, Casablanca 82403, Morocco

**Keywords:** *Helicobacter pylori*, penicillin allergy, drug therapy combination, penicillin-free regimens

## Abstract

**Background and Aims:** Amoxicillin is one of the most effective antibiotics for treating *Helicobacter pylori* infections and is widely used in first-line treatment regimens. However, patients with penicillin allergies cannot receive penicillin-based therapies, which significantly limits effective eradication options. This allergy often compels clinicians to choose alternative regimens that may be less effective, thereby increasing the risk of treatment failure. Consequently, therapeutic options for these patients are more restricted, and clinicians must carefully select the most appropriate regimen, taking into account both efficacy and the potential for antimicrobial resistance. This review aims to systematically evaluate the efficacy of penicillin-free treatment regimens for the eradication of *H. pylori* in patients with penicillin allergies. Specifically, it seeks to identify, analyze, and synthesize current clinical evidence to determine the most effective alternative therapies, thereby supporting evidence-based clinical decision-making. **Methods:** A literature search was conducted using the PubMed and Scopus databases. We began by reviewing the titles and abstracts of all identified studies to determine eligibility. Next, we assessed the full text of potentially eligible articles according to inclusion and exclusion criteria to establish the eligibility of each study. **Results:** This review included 26 studies comprising 2713 participants, evaluating penicillin-free therapies for *H. pylori* eradication in penicillin-allergic patients. Key findings demonstrated high eradication rates with bismuth-based quadruple therapies (88–97%), doxycycline-based regimens (86%), and quinolone-based therapies (75–100%), with Sitafloxacin exceeding 90% efficacy. Minocycline-based regimens also showed promising outcomes, with eradication rates between 80% and 85%. Although the PPI–clarithromycin–metronidazole combination was moderately effective, it was less favored as a first-line option. Overall, bismuth-based and quinolone-based therapies emerged as the most effective alternatives. **Conclusions:** In patients allergic to penicillin, bismuth quadruple therapy has demonstrated an excellent rate of eradication. Quinolone-based regimens are emerging as a promising alternative in first-line treatment or in cases of treatment failure. Vonoprazan-based therapy is an effective regimen. Combined with clarithromycin and metronidazole, vonoprazan enhances eradication rates and demonstrates effectiveness, including in clarithromycin-resistant strains.

## 1. Introduction

The Gram-negative, spiral-shaped bacterium *Helicobacter pylori* (*H. pylori*) is a widespread and prevalent opportunistic pathogen, most commonly associated with gastritis, peptic ulcers, and various other gastrointestinal disorders. *H. pylori* infects approximately 50–70% of the global population, with prevalence varying by geography, ethnicity, age, and socioeconomic factors [1]. *H. pylori* infection is often asymptomatic, with a majority of infected individuals (estimated between 70% and 90%) showing no clinical signs [2]. However, in a significant proportion of cases, it leads to chronic gastritis and peptic ulcer disease [1,3]. Moreover, *H. pylori* is recognized as a class I carcinogen by the World Health Organization due to its well-established role in the pathogenesis of gastric adenocarcinoma, which develops in approximately 1% of infected individuals [1]. It is also the primary etiological agent of gastric mucosa-associated lymphoid tissue (MALT) lymphoma [1,3]. Beyond these severe complications, *H. pylori* has been associated with a range of extra-gastric manifestations, including iron-deficiency anemia, idiopathic thrombocytopenic purpura, and vitamin B12 deficiency [3]. These clinical consequences underscore the importance of prompt and effective eradication strategies. The treatment is required due to the long-term complications associated with untreated infection, such as gastritis, gastric ulcers, and malignancies [3]. The goal of *H. pylori* eradication is to cure peptic ulcer disease and reduce the lifetime risk of gastric cancer [2]. Amoxicillin is one of the most effective antimicrobial agents against *H. pylori*, and therefore, most eradication regimens include this antibiotic [4]. Penicillin allergy is frequently reported, affecting approximately 10% of patients, although true IgE-mediated hypersensitivity reactions are confirmed in fewer than 1% of cases. The allergy may be immediate or delayed, with clinical manifestations ranging from mild skin rashes to severe reactions [5,6]. In clinical practice, clinicians often avoid all β-lactam antibiotics, including amoxicillin, even in the absence of confirmatory testing. This limits therapeutic options and may lead to the use of broader-spectrum antibiotics, increasing the risk of antimicrobial resistance. Therefore, it is essential to tailor *H. pylori* eradication strategies while taking this limitation into account [5]. The standard clarithromycin-based triple therapy, with metronidazole replacing amoxicillin, has been commonly used [6]. However, there is growing concern regarding the efficacy of clarithromycin triple therapy, and current guidelines no longer recommend it as a first-line treatment option [7]. In clinical practice, initial eradication therapy—referred to as first-line therapy—generally offers the highest chance of treatment success. Studies have shown that first-line regimens containing amoxicillin achieve eradication rates exceeding 90% in many cases, making them the preferred choice when no allergy is present [8]. Therefore, selecting the most appropriate first-line treatment is crucial for optimizing patient outcomes. Several studies have evaluated different first-line strategies, including standard bismuth-based quadruple therapy, modified bismuth regimens with varying antibiotic combinations, and fluoroquinolone-containing therapies, demonstrating varying degrees of efficacy [9,10,11,12]. However, in penicillin-allergic patients, choosing an effective alternative remains a clinical challenge. While bismuth-based quadruple therapies are widely recommended, their eradication rates have been inconsistent across studies, and fluoroquinolone-based regimens have shown variable efficacy depending on resistance patterns. The need for reliable alternatives is further emphasized by studies indicating that some rescue therapies fail to achieve optimal eradication rates, particularly in the context of antibiotic resistance.

Therefore, identifying the most effective treatment for persistent *H. pylori* infection in penicillin-allergic patients remains a pressing issue for gastroenterologists [13]. In this systematic review, we analyzed existing studies on penicillin-free therapies to evaluate their clinical effectiveness in both first-line and rescue treatments for *H. pylori*. Given the lack of standardized eradication guidelines in Morocco and the need for alternative regimens in penicillin-allergic patients, this review aims to provide valuable insights into the success rates and practical application of various treatment options, including bismuth-based quadruple therapies, quinolone-based regimens, and tetracycline alternatives. By addressing the challenges posed by antibiotic resistance, this study seeks to assist clinicians in selecting the most effective and safest therapies for optimal patient outcomes.

## 2. Methods

### 2.1. Search Strategy

A literature search was conducted in the PubMed and Scopus databases from 2005 to 2023 to identify studies that explored therapeutic options for patients allergic to amoxicillin with *H. pylori* infection. To obtain relevant results, the following keywords were identified: *Helicobacter pylori*, treatment, drug therapy, antibacterial agents, penicillin, amoxicillin, and allergy. The boolean terms OR, AND, and NOT were added to gather relevant articles and were used as follows: *Helicobacter pylori* AND (treatment OR drug therapy combination OR antibacterial agents) AND (penicillin OR amoxicillin) AND allergy.

In PubMed, the following Medical Subject Headings (Mesh) key terms were used: (“*Helicobacter pylori*” [MeSH Terms] OR (“*Helicobacter*” [All Fields] AND “*pylori*” [All Fields]) OR “*Helicobacter pylori*” [All Fields]) AND (“therapeutics”[MeSH Terms] OR “therapeutics” [All Fields] OR “treatments” [All Fields] OR “therapy” [MeSH Subheading] OR “therapy” [All Fields] OR “treatment” [All Fields] OR “treatment s” [All Fields] OR (“drug therapy, combination” [MeSH Terms] OR (“drug”[All Fields] AND “therapy” [All Fields] AND “combination” [All Fields]) OR “combination drug therapy” [All Fields] OR (“drug”[All Fields] AND “therapy” [All Fields] AND “combination” [All Fields]) OR “drug therapy combination” [All Fields]) OR (“anti-bacterial agents” [Pharmacological Action] OR “anti-bacterial agents” [MeSH Terms] OR (“anti-bacterial” [All Fields] AND “agents” [All Fields]) OR “anti-bacterial agents” [All Fields] OR (“antibacterial” [All Fields] AND “agents” [All Fields]) OR “antibacterial agents” [All Fields])) AND (“benzylpenicillins” [All Fields] OR “penicillin g” [MeSH Terms] OR “penicillin g” [All Fields] OR “benzylpenicillin” [All Fields] OR “penicilline” [All Fields] OR “penicillines” [All Fields] OR “penicillins” [MeSH Terms] OR “penicillins” [All Fields] OR “penicillin” [All Fields] OR (“amoxicillin” [MeSH Terms] OR “amoxicillin” [All Fields] OR “amoxicilline” [All Fields] OR “amoxicillins” [All Fields])) AND (“allergie” [All Fields] OR “hypersensitivity” [MeSH Terms] OR “hypersensitivity” [All Fields] OR “allergies” [All Fields] OR “allergy” [All Fields] OR “allergy and immunology”[MeSH Terms] OR (“allergy” [All Fields] AND “immunology” [All Fields]) OR “allergy and immunology” [All Fields]).

This transparent and reproducible approach aims to identify and select literature to produce an exhaustive analysis and critical synthesis. All suitable published papers were identified and cataloged using the bibliographic management software Zotero 6.0.30.

### 2.2. Study Selection

The references were analyzed according to predefined inclusion and exclusion criteria to determine the eligibility of each study. The selection criteria are described to ensure transparency and facilitate the objective screening of the literature. The search strategy was applied as follows: initially, titles and abstracts of all identified studies were screened for eligibility. This was followed by a full-text assessment of articles considered potentially eligible. Studies that did not meet the inclusion criteria were excluded.

Inclusion criteria:

1. Study design: Prospective, retrospective, cross-sectional, or case-control studies.
2. Language: Studies written in English only.
3. Participants:
  - All age groups, regardless of gender.
  - Patients diagnosed with *H. pylori* infection, with or without penicillin allergy.
4. Intervention:
  - First-line and/or rescue therapies.
  - Regimens using combinations of antimicrobial agents.
  - Penicillin-free regimens.
  - Studies involving clinical trials.
5. Outcome: Studies reporting on the effectiveness of *H. pylori* eradication therapy.

Exclusion criteria:

1. Study design: Non-eligible publication types such as review articles, systematic reviews, or meta-analyses.
2. Intervention:
  - Regimens without antibiotic combinations (i.e., monotherapies).
  - Studies evaluating only penicillin-containing therapies.
  - Studies without clinical trials (e.g., in vitro or animal research).
3. Outcome: Studies not reporting the effectiveness of *H. pylori* eradication therapy or studies reporting only on the efficacy of probiotics.
4. Duplicate records.

Data Extraction
The extracted data included the following:

- Study characteristics: study name, year of publication, study type, and country.
- Participant characteristics: participant age, number of subjects enrolled, and prevalence of penicillin allergy.
- Intervention characteristics: *H. pylori* eradication regimens including drugs, dosages, treatment duration, and eradication rates based on both intention-to-treat (ITT) and per-protocol (PP) analyses.
- Diagnostic methods used to detect *H. pylori* infection.

Risk of Bias

To assess the validity and reliability of the studies included in this systematic review, a risk of bias assessment was conducted for each study using the appropriate CASP checklist, depending on the study design. The aim of this assessment was to identify any methodological flaws or potential biases that could affect the findings. This step is essential for evaluating the strength of the evidence and understanding any limitations that may influence the conclusions.

## 3. Results

### 3.1. Literature Search

The studies published from 2005 to 2023 involved a total of 2713 subjects, with an average of 104 participants per study. Our search retrieved 106 articles from PubMed and 75 from Scopus, using keywords applied to titles and abstracts in the latter. A total of *n* = 181 publications were identified. Article selection was conducted in two stages: initial screening of titles and abstracts, followed by full-text review of eligible studies.

At the abstract screening stage, *n* = 144 articles were excluded. Among these, 35 were duplicate records, 34 were review articles, systematic reviews, or meta-analyses. The remaining articles were excluded primarily due to evaluating only penicillin-containing therapies.

For the full-text review, 37 studies were assessed as potentially eligible. Among them, 11 articles were excluded either because they evaluated monotherapies without antibiotic combinations or did not include clinical trials. Ultimately, *n* = 26 full-text articles were included in the final synthesis

**Figure 1:**
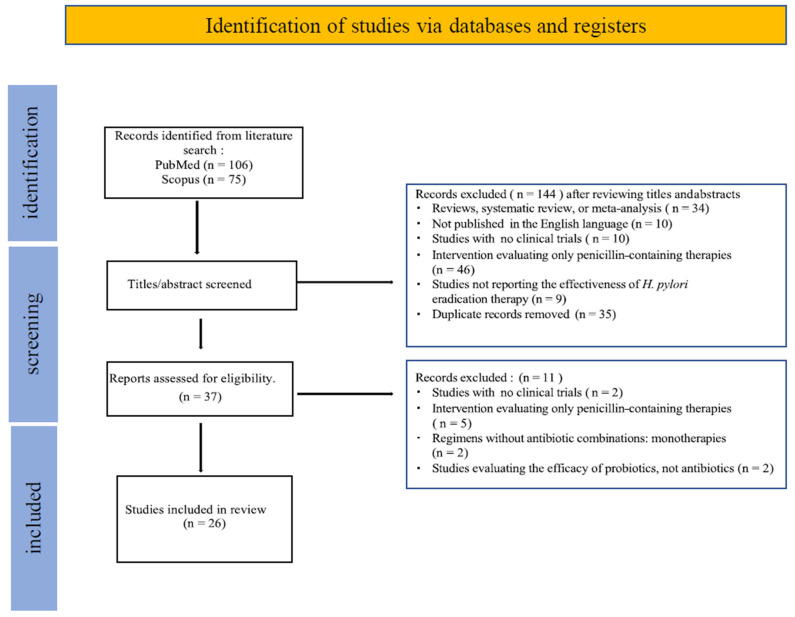
Flow diagram of study selection.

A flow diagram of the study selection and screening process is presented in Figure 1.

### 3.2. Characteristics of the Included Studies

The 26 included studies (Table 1) were conducted in various regions, predominantly in Asian countries. Among them, 19 were prospective studies, 6 were retrospective, and 1 was a case report. Participants of all age groups and both genders were included, comprising both children and adults. Treatment-naive patients were enrolled for first-line therapies, while those with prior failed eradication attempts were included in rescue regimens (second-line, third-line, or fourth-line therapies).

### 3.3. Interventions—Protocols of Treatment

Interventions consisted of penicillin-free regimens using combinations of antimicrobial agents. The identified antibiotic classes included tetracyclines (minocycline, oxytetracycline, doxycycline), 5-nitroimidazoles (metronidazole), beta-lactam cephalosporins (cefuroxime), macrolides (clarithromycin), quinolones (ciprofloxacin, sitafloxacin, levofloxacin), nitrofurans (furazolidone), and rifamycins (rifabutin). Treatment duration ranged from 7 to 10 days for short-term regimens and 14 days for long-term regimens, with 14-day treatments being the most common (10/26 studies—Table 1).

### 3.4. H. pylori Detection Methods

The assessment of *H. pylori* infection was performed by various detection methods (Table 2). Some studies diagnosed *H. pylori* infection in either invasive diagnostic tests (rapid urease test, histological analysis, culture), non-invasive diagnostic tests (13C-urea breath test (13C-UBT), *H. pylori* stool antigen test (HpSA), anti-*H. pylori* immunoglobulin G (HpIgG)), or both of them [37].

### 3.5. Risk of Bias Assessment

Appendix A summarizes the risk of bias for each study, categorized according to the key questions of the relevant CASP checklists (e.g., Cohort Study, Case-Control Study, Case Report). Each study was assessed for elements such as selection bias, measurement bias, and potential confounding factors. These assessments contribute to evaluating the overall quality of the evidence.

### 3.6. Tolerability and Compliance

Data on tolerability and compliance were available in a subset of the included studies. The most frequently reported adverse events were mild to moderate and included nausea, dizziness, headache, abdominal discomfort, and metallic taste. Minocycline-containing regimens were occasionally associated with dizziness or light-headedness, while furazolidone-based therapies were linked to gastrointestinal intolerance in some cases. Dropout rates due to adverse effects were generally low (<10%), and overall patient adherence was reported as good in most studies. However, compliance details were inconsistently reported across trials, and only a few studies provided explicit data on treatment completion rates.

## 4. Discussion

This systematic review investigates eradication strategies for *H. pylori* infection in penicillin-allergic patients and provides an overview of available penicillin-free therapies, highlighting their success rates to support therapeutic decision-making. The effective therapies are summarized in Appendix B. In Morocco, eradication regimens for *H. pylori* have not been standardized, and there is no national consensus or clinical practice guideline in place.

The Maastricht VI/Florence consensus report recommends using only regimens that achieve eradication rates ≥90% [7]. However, few currently available regimens meet this threshold. For patients allergic to amoxicillin, bismuth-based quadruple therapies represent an effective option for *H. pylori* eradication. Our study supports their use, as they show high eradication rates and include antibiotic combinations that serve as alternatives to amoxicillin. The efficacy of the PPI–bismuth–tetracycline–metronidazole quadruple therapy ranges from 88% to 97% as a first-line treatment. It can be used as an initial option for penicillin-allergic patients, as recommended by the Maastricht V and VI guidelines (2017 and 2022) [7,38]. If not used as a first-line therapy, it is widely recommended as an optimal second-line option [39]. Our review confirmed its effectiveness as a second- or third-line therapy, achieving eradication rates ≥ 90% [21,40,41].

Bismuth-based quadruple therapies containing cefuroxime have demonstrated eradication rates exceeding 80%, as reported in recent studies (2023, 2020, and 2019) [23,42,43]. Cefuroxime, a second-generation cephalosporin, is a β-lactam antibiotic with low cross-reactivity to penicillin. Nevertheless, its use in penicillin-allergic patients remains limited due to persistent concerns [44]. Cross-reactivity between penicillins and cephalosporins is primarily attributed to structural similarities, particularly in the beta-lactam ring and the side chains. These shared molecular features can be recognized by the immune system and trigger allergic responses in sensitized individuals. However, second-, third-, and fourth-generation cephalosporins often have distinct side chains, which significantly reduce the risk of cross-reactivity, making them potentially safe options for penicillin-allergic patients [44].

A recent study in 2022 evaluated the efficacy of PPI–bismuth–doxycycline–furazolidone quadruple therapy as a first-line regimen, reporting an eradication rate of 86%—comparable to 85% achieved with PPI–bismuth–amoxicillin–furazolidone [45]. This supports the potential use of doxycycline as an alternative to amoxicillin in penicillin-allergic patients.

Minocycline, a second-generation semi-synthetic tetracycline, has been underutilized in *H. pylori* treatment. Our review found studies demonstrating its potential: PPI–bismuth–minocycline–metronidazole achieved around 80% efficacy as a first-line regimen [14,22], while PPI–minocycline–metronidazole reached approximately 85% in second-line use. As an alternative to tetracycline, minocycline offers a longer half-life, allowing for once- or twice-daily dosing, compared to four times daily for tetracycline [45], thus reducing the complexity of the treatment schedule. Minocycline is generally well tolerated, but its combination with metronidazole requires close monitoring due to increased incidence of side effects such as dizziness and migraines [46].

Quinolone-based regimens (e.g., levofloxacin and sitafloxacin) represent a promising strategy for *H. pylori* eradication in penicillin-allergic patients. Studies in our review confirmed their efficacy as both first-line and rescue therapies [18,23,30], consistent with Maastricht V/VI guidelines (2017, 2020), which support their use as second-line options [7,38].

Levofloxacin, a third-generation fluoroquinolone, has demonstrated strong in vitro activity against *H. pylori* [32,47]. Eradication rates in our review ranged from 80% to 100% in first-line treatment [20,21], around 75% in second-line [21,32,41], and up to 100% in fourth-line regimens [35,41].

Sitafloxacin, a fourth-generation fluoroquinolone, achieved eradication rates above 90% in both first-line and rescue settings, with no significant difference between short- and long-term therapies. Due to the risk of serious adverse effects, the Maastricht VI/Florence consensus (2017) recommends using fluoroquinolones only when the benefits outweigh the risks [7]. Resistance to quinolones can be easily acquired, and in countries with high quinolone consumption, resistance rates are notably high [47,48].

Mori et al. evaluated the efficacy of PPI–metronidazole–sitafloxacin and found that *H. pylori* resistance to sitafloxacin did not significantly reduce eradication rates, achieving a 95% success rate [25]. The ACG Clinical Guideline advises avoiding antibiotics previously used in failed eradication regimens to prevent resistance and treatment failure [8]. However, our review included a clinical case where levofloxacin-based therapy successfully eradicated *H. pylori* in a patient with two prior treatment failures using the same fluoroquinolone [47], suggesting that, in the absence of resistance, antibiotics may still be reused.

Triple therapy with PPI–clarithromycin–amoxicillin remains a standard treatment for *H. pylori*, achieving approximately 90% eradication [49,50]. In penicillin-allergic patients, guidelines recommend replacing amoxicillin with metronidazole [3,7]. However, our findings indicate that the PPI-clarithromycin-metronidazole regimen, while moderately effective, is not the most suitable first-line option. Some studies reported improved efficacy through the following:

- Adding bismuth to the regimen, significantly increasing eradication rates.
- Extending treatment to 14 days, provided *H. pylori* is sensitive to clarithromycin and metronidazole.
- Increasing metronidazole dosage, though this may reduce adherence due to side effects.
- Substituting PPIs with vonoprazan, a novel acid suppressant that has shown promising results but is not yet available in all countries.

Despite the strength of the evidence, this review has limitations. Variability in study design, sample sizes, and geographic distribution may introduce heterogeneity, limiting comparability. Regional differences in resistance patterns also affect eradication rates. A major limitation is the lack of standardized resistance testing in clinical practice, forcing clinicians to prescribe empirically rather than based on individual susceptibility.

It is important to note that the strength of the efficacy data varies depending on the number of patients included in each treatment group. While the majority of regimens analyzed in this review were supported by sample sizes exceeding 30 patients—providing a reasonably solid basis for interpreting eradication rates—some regimens were tested in small cohorts. In particular, a few therapies were evaluated in fewer than 10 patients, which significantly limits the generalizability of their reported outcomes. For this reason, these results should be considered exploratory and interpreted with caution. For a reliable evaluation of treatment efficacy, readers are referred to Table 1, where the number of patients is clearly indicated for each regimen summarized in Appendix B.

Moreover, only studies indexed in PubMed and Scopus were included, potentially excluding relevant data from other sources. Selection bias may also be present due to the underreporting of negative findings.

Risk of bias assessment showed that 17 of the 26 included studies had a low risk of bias, indicating reliable and valid findings with strong methodology, clear study populations, and appropriate control for confounding factors.

- Eight studies had a moderate risk of bias, meaning their results are useful but should be interpreted cautiously due to some methodological limitations.
- One study was classified as high risk, with significant methodological issues that may compromise the reliability of its conclusions.

The absence of standardized *H. pylori* eradication protocols in Morocco underscores the urgent need for a national consensus. Given the high efficacy of bismuth-based and quinolone-based regimens, their inclusion in future national guidelines should be considered, alongside effective antibiotic stewardship policies to prevent resistance. Future research should prioritize large-scale randomized controlled trials (RCTs) comparing different penicillin-free regimens across various regions, with particular attention to resistance patterns. The safety and efficacy of vonoprazan-based regimens and the potential use of second-generation cephalosporins in penicillin-allergic patients should also be further explored.

This review includes numerous clinical trials with various antibiotic combinations and highlights emerging therapies, providing a valuable reference for future clinical research.

## 5. Conclusions

This systematic review provides a comprehensive synthesis of the literature on the efficacy of penicillin-free therapies used in both first-line and rescue treatments for *H. pylori* infection. The effectiveness of these regimens has been demonstrated, particularly for bismuth-based quadruple therapies, which consistently achieve high eradication rates [49,51]. The triple therapy combining PPI, metronidazole, and minocycline also appears to be a promising option, especially given its excellent eradication rate in second-line treatment. Regimens containing levofloxacin or sitafloxacin offer encouraging alternatives for both first-line use and in cases of treatment failure.

Given that a significant proportion of the included studies present a low risk of bias, the findings of this review can be considered reliable and well-supported. Nonetheless, the presence of studies with moderate or high risk of bias underscores the need to interpret results in light of study quality and to take methodological limitations into account when drawing conclusions or formulating recommendations for future research.

## Figures and Tables

**Table 1:** Overview of strategies for eradicating *H. pylori* in patients allergic to penicillin across the 26 studies in the review.

Study	Year	Country	Study Design	Age (Years)	(n) *	Treatment Details	(n) * of Each Regimen	Success Rate (PP%)	Success Rate (ITT%)
[14]	2023	China	Prospective	18–70	450	1st line MI 100 mg b.i.d., M 400 mg q.i.d., B 220 mg b.i.d., E 20 mg b.i.d., 14 days	150	91.7	84.0
						1st line MI 100 mg b.i.d., Cef 500 mg b.i.d., B 220 mg b.i.d., E 20 mg b.i.d., 14 days	150	90.9	82.7
						1st line Cef 500 mg b.i.d., M 400 mg q.i.d., B 220 mg b.i.d., E 20 mg b.i.d., 14 days	150	88.2	82.0
[15]	2023	Japan	Retrospective	38–73	53	1st line PPI (E 20 mg, L 30 mg, or R 10 mg) b.i.d., C 200 mg b.i.d., M 250 mg b.i.d., 7 days	8	50	50
						1st line V 20 mg b.i.d., C 200 mg b.i.d., M 250 mg b.i.d., 7 days	35	100	94.3
						1st line: clarithromycin resistance V 20 mg b.i.d., C 200 mg b.i.d., M 250 mg b.i.d., 7 days	11	100	90.9
						1st line or 2nd line V 20 mg b.i.d., S 50 mg b.i.d., M 250 mg b.i.d., 7 days	10	90	90
[16]	2022	China	Prospective	18–65	92	1st line I 5 mg b.i.d., D 100 mg b.i.d., F 100 mg b.i.d., B 220 mg b.i.d. for 14 days and I 5 mg q.i.d., for an additional 14 days	92	92.9	85.9
[17]	2021	China	Case report	47	1	4th line L 500 mg q.d., F 100 mg b.i.d., B 220 mg b.i.d., E 20 mg b.i.d., 14 days	1	N/A	N/A
[18]	2021	Japan	Prospective	49–74	17	2nd line V 20 mg b.i.d., M 250 mg b.i.d., S 100 mg b.i.d., 7 days	17	88.2	88.2
[19]	2021	Spain	Prospective	mean 56	55	1st line B, M, T (three tablets q.i.d.; one tablet is an association of the three principles), P 40 mg b.i.d., 10 days	45	N/A	95.6
						2nd line B, M, T (three tablets q.i.d.; one tablet is an association of the three principles), P 40 mg b.i.d., 10 days	6	N/A	100
						3rd line B, M, T (three tablets q.i.d.; one tablet is an association of the three principles), P 40 mg b.i.d., 10 days	4	N/A	50
[12]	2020	China	Prospective	8–13	22	1st line O 1.0 mg/kg/day q.d. or b.i.d., C 20 mg/kg/day b.i.d., M 20 mg/kg/day b.i.d., or t.i.d., B 6–8 mg/kg/day b.i.d., 14 days	22	N/A	90.9
[20]	2020	China	Prospective	21–73	112	1st line E 20 mg b.i.d., C 500 mg b.i.d., M 400 mg b.i.d., 14 days	5	100	100
						1st line E 20 mg b.i.d., LF 500 mg q.d., M 400 mg b.i.d., 14 days	1	100	100
						1st and 2nd line E 20 mg b.i.d., T 500 mg q.i.d., M 400 mg b.i.d., 14 days	2	100	100
						1st and 2nd line E 20 mg b.i.d., B 220 mg b.i.d., C 500 mg b.i.d., M 400 mg q.i.d., 14 days	22	94.1	81.8
						1st and 2nd line E 20 mg b.i.d., B 220 mg b.i.d, LF 500 mg q.d., M 400 mg q.i.d., 14 days	10	100	80
						1st and 2nd line E 20 mg b.i.d., B 220 mg b.i.d, T 500 mg q.i.d., M 400 mg q.i.d., 14 days	72	100	97.2
[21]	2020	Europe	Prospective	38–68	562	(Drug dose, frequency, and duration not specified) 1st line—PPI, C, M;	228	69	69
						1st line—PPI, C, LF	50	82	80
						1st line—PPI, B, T, M	228	92	91
						2nd line—(failed PPI, C, M) PPI, C, LF	17	69	71
						2nd line—(failed PPI, C, M) PPI, M, LF	13	77	77
						2nd line—(failed PPI, C, M) PPI, B, T, M	64	82	78
						2nd line—(failed PPI, C, LF) PPI, B, T, M	5	80	80
						2nd line—(failed PPI, B, T, M) PPI, C, LF	3	100	100
						2nd line—(failed PPI, B, T, M) PPI, M, LF	4	75	75
						3rd line—(failed PPI, C, M and then PPI, C, LF) PPI, B, T, M	12	82	75
						3rd line—(failed PPI, C, M and then PPI, M, LF) PPI, B, T, M	5	100	100
						3rd line—(failed PPI, C, M and then PPI, B, T, M) PPI, C, LF	2	50	50
						3rd line—(failed PPI, C, LF) Repeated PPI, B, T, M	1	0	0
						3rd line—(failed PPI, B, T, M and PPI, M, LF) PPI, C, M, LF	1	100	100
[22]	2019	China	Prospective	18–70	118	1st line R 10 mg b.i.d., Mi 1000 mg b.i.d., M 400 mg t.i.d., B 220 mg b.i.d., 14 days	118	84.3	77.1
[23]	2019	China	Prospective	29–57	152	1st line Cef 500 mg b.i.d., LF 500 mg q.d., E 20 mg b.i.d., B 220 mg b.i.d., 14 days	152	90.1	85.5
[11]	2018	China	Prospective	25–65	66	1st line E 20 mg b.i.d., C 500 mg b.i.d., M 400 mg q.i.d., 14 days	33	70	63.6
						1st line E 20 mg b.i.d, C 500 mg b.i.d., M 400 mg q.i.d., B 600 mg b.i.d., 14 days	33	96	84.8
[24]	2017	Japan	Prospective	58–79	50	1st line V 20 mg b.i.d., C 200 or 400 mg b.i.d., M 250 mg b.i.d., 7 days	20	100	100
						1st line PPI (L 30 mg b.i.d. or E 20 mg b.i.d.), C 200 or 400 mg b.i.d., M 750 mg 7 days	30	82.7	83.3
[25]	2017	Japan	Prospective	44–72	57	1st line E 20 mg b.i.d., SF 100 mg b.i.d., M 250 mg b.i.d., 10 days	33	100	100
						2nd line E 20 mg b.i.d., SF 100 mg b.i.d., M 250 mg b.i.d., 10 days	19	84.2	84.2
						3rd line E 20 mg b.i.d., SF 100 mg b.i.d., M 250 mg b.i.d., 10 days	5	40	40
[26]	2017	Japan	Retrospective	mean 59	88	1st line PPI (L 30 mg or R 20 mg) b.i.d., C 200 mg b.i.d., M 250 mg b.i.d., 7 days	10	55.6	50
						2nd line PPI b.i.d., C 200 mg b.i.d., M 250 mg b.i.d., 7 days	3	33.3	33.3
						1st line V 20 mg b.i.d., C 200 mg b.i.d., M 250 mg b.i.d., 7 days	13	92.3	92.3
						2nd line V 20 mg b.i.d., C 200 mg b.i.d., M 250 mg b.i.d., 7 days	1	100	100
						1st line PPI b.i.d., SF 100 mg b.i.d., M 250 mg b.i.d., 7 days	20	100	100
						2nd line PPI b.i.d., SF 100 mg b.i.d., M 250 mg b.i.d., 7 days	24	100	100
						1st line V 20 mg b.i.d., SF 100 mg b.i.d., M 250 mg b.i.d., 7 days	14	100	100
						2nd line V 20 mg b.i.d., SF 100 mg b.i.d., M 250 mg b.i.d., 7 days	3	66.7	66.7
[27]	2017	Turkey	Prospective	32–58	111	1st line R 20 mg b.i.d., B 562 mg b.i.d., M 500 mg t.i.d., T 500 mg q.i.d., 10 days	111	92.5	88.3
[28]	2017	Japan	Retrospective	26–83	5	2nd line R 20 mg b.i.d., 250 mg M b.i.d., 100 mg MI b.i.d., 7 days	5	N/A	100
[29]	2016	China	Prospective	24–76	156	3rd line or later line: L 30 mg b.i.d., B 220 mg b.i.d., M 400 mg q.i.d., T 500 mg q.i.d., 14 days	156	95.3	87.2
[4]	2015	Spain	Prospective	mean 52	267	1st line O 20 mg b.i.d., C 500 mg b.i.d., M 500 mg b.i.d., 7 days	112	59	57
						1st line O 20 mg b.i.d., B 120 mg q.i.d., T (Oxy 500 mg q.i.d. or D 100 mg b.i.d.), M 500 mg t.i.d., 10 days	50	75	74
						2nd line (after failed OCM) O 20 mg b.i.d., B 120 mg q.i.d., T (Oxy 500 mg q.i.d. or D 100 mg b.i.d.), M 500 mg t.i.d., 10 days	24	38	37
						2nd line (after failed OCM) O 20 mg b.i.d., C 500 mg b.i.d., LF 500 mg b.i.d., 10 days	50	73	64
						2nd line (after failed OBTM) O 20 mg b.i.d., C 500 mg b.i.d., LF 500 mg b.i.d., 10 days	14	64	64
						3rd line O 20 mg b.i.d., C 500 mg b.i.d., LF 500 mg b.i.d., 10 days	3	50	33
						3rd line O 20 mg b.i.d., C 500 mg b.i.d., RIF 150 mg b.i.d., 10 days	7	20	14
						3rd line O 20 mg b.i.d., B 120 mg q.i.d., T 500 mg q.i.d., M 500 mg t.i.d., 10 days	3	100	100
						4th line O 20 mg b.i.d., C 500 mg b.i.d., RIF 150 mg b.i.d., 10 days	2	0	50
						4th line O 20 mg b.i.d., C 500 mg b.i.d., LF 500 mg b.i.d., 10 days	2	100	100
[30]	2014	Japan	Retrospective	46–68	28	1st line PPI (R 10 mg b.i.d., or L 30 mg b.i.d., or E 20 mg b.i.d.), SF 100 mg b.i.d., M 250 mg b.i.d., 7 days	7	100	100
						1st line PPI (R 10 mg b.i.d., or q.i.d; E 20 mg b.i.d.), SF 100 mg b.i.d., M 250 mg b.i.d., 14 days	4	100	100
						2nd line PPI (R 10 mg b.i.d., or q.i.d; or E 20 mg b.i.d.), SF 100 mg b.i.d., M 250 mg b.i.d., 7 days	9	100	100
						2nd line PPI (R 10 mg b.i.d., or q.i.d; or E 20 mg b.i.d.), SF 100 mg b.i.d., M 250 mg b.i.d., 14 days	3	100	100
						3rd line PPI (R 10 mg b.i.d., or q.i.d; or E 20 mg b.i.d.), SF 100 mg b.i.d., M 250 mg b.i.d., 7 days	3	100	100
						3rd line PPI (R 10 mg b.i.d., or q.i.d; or E 20 mg b.i.d.), SF 100 mg b.i.d., M 250 mg b.i.d., 14 days	2	100	100
[31]	2012	Australia	Retrospective	16–85	69	2nd line R 20 mg t.i.d., B 240 mg q.i.d., RIF 150 mg b.i.d., CF 500 mg b.i.d., 10 days	69	94.2	94.2
[32]	2010	Spain	Prospective	33–69	50	1st line O 20 mg b.i.d., C 500 mg b.i.d., M 500 mg b.i.d., 7 days.	50	55	54
						2nd line O 20 mg b.i.d., C 500 mg b.i.d., LF 500 mg b.i.d., 10 days.	15 out of the 50	73	73
[33]	2006	Japan	Prospective	41–63	67	2nd line R 20 mg b.i.d., MI 100 mg b.i.d., M 250 mg b.i.d., 7 days	67	N/A	85.1
[34]	2006	Japan	Retrospective	40–64	5	1st line PPI (L 30 mg, O 20 mg or R 10 mg q.d.), T 500 mg b.i.d., M 250 mg b.i.d., 7-14 days	5	100	80
[35]	2005	Spain	Prospective	mean 57	40	1st line O 20 mg b.i.d., C 500 mg b.i.d., M 500 mg b.i.d., 7 days	12	64	58
						2nd line RBC 400 mg b.i.d., T 500 mg q.i.d., M 250 mg q.i.d., 7 days	17	53	47
						3rd line O 20 mg b.i.d., C 500 mg b.i.d., RIF 150 mg b.i.d., 10 days	9	17	11
						4th line O 20 mg b.i.d., C 500 mg b.i.d., LF 500 mg b.i.d., 10 days	2	100	100
[36]	2005	Puerto Rico	Prospective	mean 59	20	1st line E 40 mg q.i.d., T 500 mg q.i.d., M 500 mg q.i.d., 10 days	17	N/A	84
						2nd line E 40 mg q.i.d., T 500 mg q.i.d., M 500 mg q.i.d., 10 days	3	N/A	100

Abbreviations: (n) *: number of participants; PP: Per protocol analysis; ITT: Intention to treat analysis; q.d.: once a day; b.i.d.: twice a day; t.i.d.: three times a day; q.i.d.: four times a day; N/A: not available. B: Bismuth; C: Clarithromycin; Cef: Cefuroxime; CF: Ciprofloxacin; D: Doxycycline; E: Esomeprazole; F: Furazolidone; I: Ilaprazole; L: lansoprazole; LF: Levofloxacin; M: Metronidazole; Mi: Minocycline; O: Omeprazole; Oxy: Oxytetracycline; R: Rabeprazole; RBC: Ranitidine Bismuth Subcitrate; RIF: Rifabutin; P: Pantoprazole; PPI: Proton Pump Inhibitor; SF: Sitafloxacin; T: Tetracycline; V: Vonoprazan.

**Table 2:** Overview of the diagnostic methods for *H. pylori* utilized in the 26 reviewed studies.

Study	Year	Country	Study Design	Methods of Diagnosis
[14]	2023	China	Prospective	Diagnostic positive in the following: - 13C-UBT or positive in both Rapid urease test and Histology
[15]	2023	Japan	Retrospective	Diagnostic positive in one of the four tests: - 13C-UBT, Rapid urease test, Histology, Culture
[16]	2022	China	Prospective	Diagnostic positive in one of the two tests: - 13C-UBT, Histology
[17]	2021	China	Case report	Diagnostic positive in Histology
[18]	2021	Japan	Prospective	Diagnostic positive in one of the five tests: - 13C-UBT, HpIgG, Histology, Culture, SAT
[19]	2021	Spain	Prospective	Diagnostic positive in both: - Rapid urease test and Culture
[12]	2020	China	Prospective	Diagnostic positive in the following: - Culture or positive in both Rapid urease test and Histology
[20]	2020	China	Prospective	Diagnostic positive in one of the two tests: - 13C-UBT, Rapid urease test, or Histology
[21]	2020	Europe	Prospective	Diagnostic positive in one of the five tests: - 13C-UBT, Histology, Culture, SAT, Rapid urease test
[22]	2019	China	Prospective	Diagnostic positive in one of the two tests: - 13C-UBT and both Rapid urease test and Histology
[23]	2019	China	Prospective	Diagnostic positive in the three tests: - 13C-UBT, Rapid urease test, Histology
[11]	2018	China	Prospective	Diagnostic positive in the following: - 13C-UBT and at least one of the three tests: - Rapid urease test, Histology, Culture
[24]	2017	Japan	Prospective	Diagnostic positive in one of the five tests: - 13C-UBT, HpIgG, Histology, Culture, Rapid urease test
[25]	2017	Japan	Prospective	Diagnostic positive in one of the two tests: - 13C-UBT, SAT
[26]	2017	Japan	Retrospective	Diagnostic positive in 13C-UBT
[27]	2017	Turkey	Prospective	Diagnostic positive in Histology
[28]	2017	Japan	Retrospective	Diagnostic positive in the three tests: - 13C-UBT, HpIgG, Histology
[29]	2016	China	Prospective	Diagnostic positive in the following: - 13C-UBT and at least one of the three tests: - Rapid urease test, Histology, Culture
[4]	2015	Spain	Prospective	Diagnostic positive in the three tests: - 13C-UBT, Rapid urease test, Histology
[30]	2014	Japan	Retrospective	Diagnostic positive in the two tests: - Rapid urease test and Culture
[31]	2012	Australia	Retrospective	Diagnostic positive in one of the two tests: - Culture, Histology
[32]	2010	Spain	Prospective	Diagnostic positive in one of the three tests: - 13C-UBT, Rapid urease test, Histology
[33]	2006	Japan	Prospective	Diagnostic positive in: Rapid urease test
[34]	2006	Japan	Retrospective	Diagnostic positive in one of the three tests: - 13C-UBT, Culture, Histology
[35]	2005	Spain	Prospective	Diagnostic positive in one of the three tests: - 13C-UBT, Rapid urease test, Histology
[36]	2005	Puerto Rico	Prospective	Diagnostic positive in the two tests: - Rapid urease test and Histology

Abbreviations: 13C-UBT: 13C-urea breath test; HpIgG: Anti-*H. pylori* Immunoglobulin G; SAT: Stool *H. pylori* antigen test.

## Appendix A. Summary of Risk of Bias Assessment for Included Studies

**Study ID**	**Study Type**	**Selection Bias**	**Justification**	**Measurement Bias**	**Justification**	**Confounding Bias**	**Justification**	**Overall Risk**
[14]	Randomized controlled trial	Low	Randomized groups ensure comparability	Low	Objective outcome measure (urea breath test)	Low	Confounders considered, compliance assessed	Low
[15]	Retrospective	Moderate	Not randomized, selection bias possible due to patient selection.	Low	13C-UBT breath test is a reliable outcome measure.	Moderate	Small sample size (n = 53), potential confounders not fully controlled.	Moderate
[16]	RCT	Low	Randomized multicenter trial reduces selection bias.	Low	Objective measures (eradication rates, ulcer healing).	Low	Balanced groups, no major confounders noted.	Low
[17]	Case Report	High	Single-patient case, no randomization.	Moderate	Use of agar dilution susceptibility testing ensures reliability, but single case limits generalizability.	High	No control group, high risk of confounding.	High
[18]	Prospective Intervention	Moderate	Small sample size (n = 17), limited generalizability.	Low	Objective measures used for eradication confirmation.	Moderate	Lack of control group increases confounding risk.	Moderate
[19]	RCT	Low	Randomized trial comparing AST-guided therapy vs. Pylera^®^.	Low	Objective outcome measure (fecal antigen test).	Low	Balanced groups, adherence and side effects analyzed.	Low
[12]	Prospective Cohort	Moderate	Not randomized, selection bias possible.	Low	Objective microbiome analysis via 16S rRNA gene sequencing.	Moderate	No control over external factors affecting microbiome.	Moderate
[20]	Randomized controlled trial	Moderate	No mention of allocation concealment, which could lead to selection bias.	Low	The E-test method for antimicrobial susceptibility is a standard and reliable measurement.	Low	Randomization controls confounding factors, and the populations were comparable.	Low
[21]	Registry-based study	Moderate	No randomization, potential for selection bias in choosing patients from the registry.	Low	The methodology and efficacy outcomes were well-defined, with good validation of the treatments.	Low	The large sample size and use of standard treatment regimens minimize confounding.	Moderate
[22]	Randomized controlled trial	Moderate	No mention of allocation concealment or blinding, which could lead to selection bias.	Low	The 13C-UBT breath test was used, which is a standard and reliable method for *H*. *pylori* eradication assessment.	Low	Randomization was performed, and baseline characteristics are similar, minimizing confounding.	Low
[23]	Cohort study	Moderate	No mention of randomization or allocation concealment, which could lead to selection bias.	Low	Urea breath test was used to confirm *H*. *pylori* eradication, which is reliable.	Low	The study does not have confounding variables since it is well controlled with penicillin allergy as the main criterion.	Low
[11]	Randomized controlled trial	Moderate	No mention of allocation concealment, which could lead to selection bias.	Low	13C-UBT breath test was used, which is a reliable measure for *H*. *pylori* eradication.	Low	Randomization is performed, and the two groups are comparable, minimizing confounding.	Low
[24]	Prospective observational study	Moderate	Small sample size (20 patients) may lead to selection bias.	Low	13C-UBT breath test is a reliable measure for *H*. *pylori* eradication.	Low	Random assignment of patients to VPZ-based and PPI-based regimens, minimizing confounding.	Low
[25]	Cohort study	Moderate	No randomization, and patients were not equally distributed between resistant and non-resistant groups.	Low	The use of 13C-UBT breath test and stool antigen test is reliable for confirming eradication.	Low	Resistance to antibiotics (sitafloxacin and metronidazole) could be a confounder, but the study adjusts for this.	Moderate
[26]	Retrospective cohort study	Moderate	Retrospective design may lead to selection bias.	Low	13C-UBT breath test is a reliable method for determining eradication success.	Low	The study considered different regimens and analyzed intention-to-treat vs. per-protocol outcomes, minimizing confounding.	Moderate
[27]	Randomized controlled trial	Low	Randomization of patients into 3 groups reduces selection bias.	Low	Standardized methodologies for measuring eradication and evaluating side effects.	Low	Randomization and large sample size minimize potential confounders.	Low
[28]	Cohort study	Moderate	No randomization, but a large sample size (650 patients) reduces selection bias.	Low	Serum PG I/II ratios were measured before and after treatment, which is reliable.	Low	No significant confounders mentioned, but the study acknowledges variations in treatment regimens.	Low
[29]	Randomized controlled trial	Low	Randomization reduces selection bias.	Low	Use of antimicrobial susceptibility testing and standardized eradication protocols.	Low	Randomization minimizes confounding. The study accounts for resistance factors.	Low
[4]	Prospective multicenter	Low	Consecutive patients were included, reducing the likelihood of selection bias.	Low	The use of 13C-UBT is objective and reliable for measuring eradication.	Low	No major confounders were identified; treatment regimens were standardized.	Low
[30]	Prospective multicenter	Low	28 consecutive patients were treated, reducing bias in selection.	Low	13C-UBT is used for accurate measurement of eradication.	Low	The study clearly focused on penicillin-allergic patients with standardized treatment regimens.	Low
[31]	Observational cohort	Moderate	Patients who failed previous treatments were included, potentially creating a selection bias.	Moderate	Pre-antibiotic sensitivity testing can lead to misclassification of resistance.	Moderate	Resistance to clarithromycin and metronidazole could influence results, especially in resistant strains.	Moderate
[32]	Prospective multicenter	Low	Consecutive patients with penicillin allergy were included, minimizing selection bias.	Low	13C-UBT is reliable for eradication assessment.	Low	No significant confounding factors were identified; treatment regimens were carefully managed.	Low
[33]	Randomized controlled trial	Moderate	Randomization was performed, but may have overlooked some baseline differences.	Moderate	Eradication was measured, but the distinction between metronidazole-sensitive and resistant strains could introduce some bias.	Moderate	The use of metronidazole and amoxicillin can be affected by resistance patterns, introducing potential confounding.	Moderate
[34]	Observational cohort	Low	Patients were treated consecutively, minimizing selection bias.	Low	HP eradication was tested using reliable methods, reducing measurement bias.	Low	No significant confounders were identified in the analysis, and treatment regimens were standardized.	Low
[35]	Prospective single-center	Low	Consecutive patients with penicillin allergy were included, reducing selection bias.	Low	13C-UBT used for measuring eradication is accurate and reliable.	Low	No significant confounders identified; therapies were well-defined and standardized.	Low
[36]	Prospective single-center	Low	20 consecutive patients were included, reducing selection bias.	Low	Follow-up panendoscopy and biopsies were used to ensure reliable measurement of HP eradication.	Low	No major confounders were identified; standard treatment regimen used.	Low

## Appendix B. Efficacy of Therapeutic Protocols in Case of Penicillin Allergy *

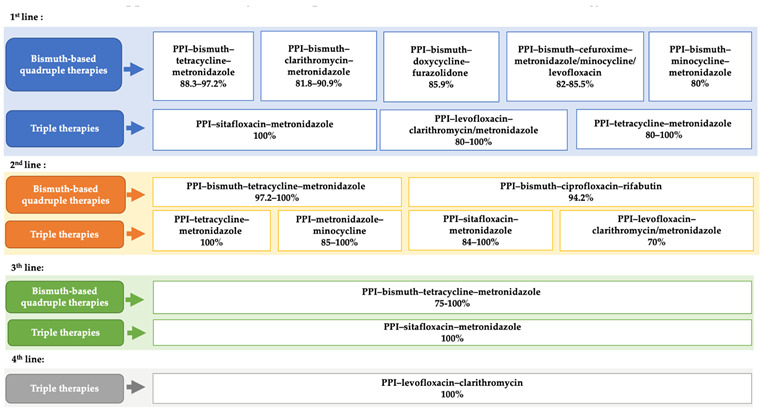

* For detailed information on individual treatment regimens, including sample sizes and eradication rates, please refer to Table 1. It is important to note that several regimens listed in this appendix are based on a small number of participants (n < 10), which may limit the reliability and generalizability of the reported efficacy outcomes.
